# Genome-Wide Associations for Microscopic Differential Somatic Cell Count and Specific Mastitis Pathogens in Holstein Cows in Compost-Bedded Pack and Cubicle Farming Systems

**DOI:** 10.3390/ani11061839

**Published:** 2021-06-21

**Authors:** Patricia Wagner, Tong Yin, Kerstin Brügemann, Petra Engel, Christina Weimann, Karen Schlez, Sven König

**Affiliations:** 1 Institute of Animal Breeding and Genetics, Justus-Liebig-University of Gießen, Ludwigstr. 21b, 35390 Giessen, Germany; tong.yin@agrar.uni-giessen.de (T.Y.); kerstin.bruegemann@agrar.uni-giessen.de (K.B.); Petra.Engel@agrar.uni-giessen.de (P.E.); Christina.Weimann@agrar.uni-giessen.de (C.W.); sven.koenig@agrar.uni-giessen.de (S.K.); 2 Landesbetrieb Hessisches Landeslabor, Schubertstraße 60, D-35392 Gießen, Germany; Karen.Schlez@lhl.hessen.de

**Keywords:** genome-wide associations, genotype by environment interaction, new free walk housing, differential somatic cell fractions, udder health

## Abstract

**Simple Summary:**

New free walk housing systems such as compost-bedded pack barns might positively influence animal welfare. However, udder health can be negatively affected due to the microbial environment in the pack. Udder health depends on many factors, such as the environment, the feed, the pathogen species, and the genetic mechanisms of the cow’s immune system. For a more precise evaluation of udder health, we examined novel traits including specific mastitis pathogens and differential somatic cell fractions in milk. In order to identify possible candidate genes for udder health, a genome-wide association study, including single-nucleotide polymorphisms (SNP) by housing system interactions (compost-bedded pack barn and conventional cubicle barn), was performed. We identified two potential candidate genes for the interaction effect in relation to udder health. The identified potential candidate gene *HEMK1* (HemK methyltransferase family member 1) is involved in immune system development, and *CHL1* (cell adhesion molecule L1 like) has an immunosuppressive effect during stress conditions. The results suggest housing system-specific breeding strategies in order to improve udder health in compost-bedded pack and conventional cubicle barns.

**Abstract:**

The aim of the present study was to detect significant SNP (single-nucleotide polymorphism) effects and to annotate potential candidate genes for novel udder health traits in two different farming systems. We focused on specific mastitis pathogens and differential somatic cell fractions from 2198 udder quarters of 537 genotyped Holstein Friesian cows. The farming systems comprised compost-bedded pack and conventional cubicle barns. We developed a computer algorithm for genome-wide association studies allowing the estimation of main SNP effects plus consideration of SNPs by farming system interactions. With regard to the main effect, 35 significant SNPs were detected on 14 different chromosomes for the cell fractions and the pathogens. Six SNPs were significant for the interaction effect with the farming system for most of the udder health traits. We inferred two possible candidate genes based on significant SNP interactions. *HEMK1* plays a role in the development of the immune system, depending on environmental stressors. *CHL1* is regulated in relation to stress level and influences immune system mechanisms. The significant interactions indicate that gene activity can fluctuate depending on environmental stressors. Phenotypically, the prevalence of mastitis indicators differed between systems, with a notably lower prevalence of minor bacterial indicators in compost systems.

## 1. Introduction

Genotype by environment interactions (GxE) in dairy cows have been reported widely [1,2,3,4]. There are many different approaches to prove GxE, such as creating a cross-classified research design for, e.g., climate or feeding groups [2], or calculating correlations between the same trait recorded in different environments [3,4,5]. Another approach focusses on continuous environmental descriptors such as temperature, herd production level, or herd size and the application of random regression or reaction norm methodology [3].

In genomic studies, Lillehammer et al. [3] and Hayes et al. [6] enhanced their genome-wide association studies (GWAS) by considering GxE effects. In a two-step approach, they estimated intercept and slope effects for each single-nucleotide polymorphism (SNP) and found environment-sensitive and environment-robust SNPs. Similarly, Streit et al. [1] conducted a GWAS for milk protein yield using average herd somatic cell score (SCS) as an environmental descriptor. Dependent variables in their genome-wide association models were intercept and slope from reaction norm models.

New animal-friendly “free walk” housing systems, such as compost farming, reduce environmental stress, implying improved animal welfare [7]. However, the open-bedded pack area, as a mixture of composting material, microbes, and feces, increases the risk for specific bacterial infections and mastitis [7,8,9]. In this regard, several studies pointed out the associations between bedding management and physiological cow responses and addressed overall immunity [7,10,11]. A possible breeding approach is the selection of mastitis-resistant animals in a challenging environment [11,12]. Selection of animals in harsh environments implied a pronounced genetic differentiation in udder health indicator traits [13]. Nevertheless, such a selection strategy, in specific environments, raises concerns regarding site suitability for conventional farming systems, and vice versa.

Furthermore, selection on mastitis resistance is hampered due to the trait definitions and the complex polygenic mode of inheritance [12,14]. Heritabilities for SCS and overall clinical mastitis were very small and lower than 0.05 [15,16,17]. Regarding accurate trait definitions, difficulties in distinguishing between cases of clinical and subclinical mastitis were reported [11,16,18]. SCS is considered a mastitis indicator in overall dairy cattle breeding goals or selection indices, due to a genetic correlation of approximately 0.75 [19]. However, the genetic correlation being substantially smaller than one suggests alternative and more precise mastitis indicators, such as differential somatic cell count, should be considered.

Previous studies indicate that mastitis is caused by different pathogens to which the cow may respond differently [11]. Sørensen et al. reported that genetic correlations between pathogen-specific mastitis traits ranged widely from 0.45 to 0.77 [14]. In their study, the lowest correlation of 0.45 was estimated between *Staphylococcus aureus* and *Escherichia coli*, possibly due to the different immune responses of these two pathogens. The highest correlation of 0.77 was detected between *Streptococcus dysgalactiae* and *Streptococcus uberis*. Both pathogens belong to the same genus and trigger a similar cascade of immune reactions in the udder [14]. The moderate genetic correlations suggest consideration that different mastitis pathogens can impact different traits in genetic evaluations for udder health. Addressing the farming effect, different bedding materials and different compost temperatures were associated with specific types of mastitis [7]. In this regard, *Coagulase-negative staphylococci* (CNS) and *Corynebacterium sp.* (COR), as well as minor pathogens, are of increasing importance [20,21]. Accordingly, due to pathogen-specific reactions of the immune system, Kurz et al. [22] suggested pathogen-specific mastitis analyses instead of the only moderately correlated indictor trait SCS. Hence, we decided to focus on novel mastitis traits in the present genomic study, including specific mastitis pathogens and differential somatic cell fractions comprising lymphocytes (LYM), macrophages (MAC), polymorphonuclear leucocytes (PMN), and their subgroups of PMN segmented neutrophils (sN) and banded neutrophils (bN), and considered these for detailed phenotypic analyses in free walk housing systems [23].

In a previous GWAS without farming interaction effect, dense SNP marker panels were used to infer possible candidate genes playing a role in defense mechanisms against pathogens and mastitis resistance [11]. Possible candidate genes or quantitative trait loci (QTL) and SNPs for clinical mastitis were detected on nearly all chromosomes [11,12], again raising the question of proper mastitis trait definitions. Nevertheless, in gene annotations, detected genes such as *LY6K, LY6D, LYNX1, LYPD2, SLURP1*, and *PSCA* are part of the lymphocyte antigen 6 complex and influence the regulation of PMN [12]. Further detected genes, e.g., *LY75, NR4A2*, and *ITGB6*, are involved in regulating immune response or were regulated during the presence of specific pathogens [12]. Kurz et al. [22] identified *RASGRP1* as a potential candidate gene based on differential gene expression studies, considering different levels of pathogen burden.

The aim of the present study was to identify significant main and interaction effects for specific mastitis pathogens and differential somatic cell counts in conventional cubicle and compost-bedded farming systems. Significant SNP markers were annotated with potential candidate genes with the aim of improving mastitis resistance in specific farming systems, taking GxE into account. In this regard, our own software package, allowing GWAS with SNP by housing system effects, was applied.

## 2. Materials and Methods

### 2.1. Animal Ethics Statement

Data for this study considered milk samples from routine milking. No additional experiments were conducted. Thus, in concordance with German animal welfare legislation, no ethical approval was required for this study.

### 2.2. Farms and Animals

Cow milk samples were collected from six selected Holstein dairy cattle herds. The herds were located in the German federal states of North Rhine-Westphalia and Hesse. Herd selection for cubicle and compost-bedded farming systems considered the herd criteria as defined in the collaborative EU FreeWalk project [24]. The case (compost) and control (cubicle) farms were identical regarding herd size, production level, climate conditions, location, and feeding and milking systems, as well as management aspects [24]. The only major difference was the bedding component. In two cases, in the same location with the same climatic conditions and geographical coordinates, and using the same milking technique and feed ration, both compost and cubicle systems were available. Three herds or sub-herds for each farming system compost and cubicle were selected.

Trait recording spanned the period from October 2018 to April 2019. The final dataset comprised milk samples from 2198 udder quarters from 537 first and second parity Holstein Friesian dairy cows. A total of 44% of the samples represented the compost system, while 56% of the samples represented the cubicle system.

### 2.3. Milk Sample Preparation and Udder Health Trait Determination

Teats were disinfected with 70% ethanol before milking and the first five squirts of milk were discarded. A quantity of 10 mL milk per udder quarter was examined for specific mastitis pathogens and somatic cell count in the laboratory of Landesbetrieb Hessen, following the DVG guidelines [25]. The specific mastitis pathogens were classified into major pathogens (MAJOR) including *Aerococcus sp., Aesculin hydrolyzing streptococci, Candida krusei, Enterococcus sp., Escherichia Coli, Lactococcus sp., Staphylococcus aureus, Streptococcus dysgalactiae, Streptococcus uberis,* mold fungus, and *Proteus sp.*; minor pathogens (MINOR) included *Coagulase-negative staphylococci* and *Corynebacterium sp*. The following pathogens were additionally analyzed separately: *Coagulase-negative staphylococci* (CNS), *Corynebacterium sp.* (COR), *Aesculin hydrolysing streptococci* (AESC), and *Aerobic bacilli* (AER). The pathogens were defined as binary traits. A cow received a score of 1 for the overall group definition (MAJOR or MINOR) or for CNS, COR, AESC, or AER when the respective pathogen was detected in at least one udder quarter, otherwise a score of 0 was assigned. At the individual cow level, 32.9% and 12.6% of all samples were culturally negative in the compost and in the cubicle system, respectively. The prevalence of minor pathogens was 57.2% in compost and 80.6% in cubicle. A fraction of 16.7% of cows kept in compost and 6.1% of cows kept in cubicle had infections with AER. The prevalence of AESC was 5.0% in compost and 4.8% in cubicle.

Microscopic differential cell counting from a 50 mL sample was conducted in the laboratory of the Institute of Animal Breeding and Genetics, University of Gießen, according to the protocols by Sarikaya et al. and Pappenheim [26,27]. We considered LYM, MAC, PMN, and the PMN subgroups sN and bN. The sum of these cell fractions was 100%. The individual cell fractions were defined as percentages in the subsequent analyses. Quarters with less than 30 counted cells were excluded from the study. Mean values for LYM were 82.0% in compost and 79.4% in cubicle. The mean value for MAC in compost was 47.6% and 49.4% in cubicle. Mean values for PMN were 21.6% in compost and 20.4% in cubicle.

### 2.4. Cow Genotyping

The 273 Holstein cows from both systems were genotyped based on extracted DNA from hair samples in the laboratory of the veterinary institute (The Center for Molecular Diagnostics), Georg-August-Universität Göttingen, using the Illumina BovineSNP50 Bead Chip V3. The SNP genotypes from the Illumina BovineSNP50 Bead Chip V2 for the 277 cows (compost and cubicle) were provided by the Vereinigte Informationssysteme Tierhaltung w.V. (VIT). A total of 53,217 SNPs from Bead Chip V3 and 45,613 SNPs from Bead Chip V2 were available for quality control. In this regard, only the SNP available in both genotype datasets and located on Bos taurus autosomes were considered. The SNP positions were remapped according to the ARS1.2 assembly [28]. Quality control was performed using PLINK software, version 1.9 [29]. The SNP markers with a minor allele frequency <0.01, a minimum call rate <90%, and which were in Hardy–Weinberg equilibrium (*p* ≥ 10^−3^) were discarded. After quality control, we considered 43,095 SNPs from 550 genotyped cows (235 genotyped cows from compost and 315 genotyped cows from cubicle). For the ongoing GWAS, 537 cows with genotypes and phenotypic information were included.

### 2.5. Genome-Wide Associations

Single-trait GWAS with interaction effects between farming systems and SNP effects were conducted using the self-written R program “GWAInter.R”. All relevant data and the program source code are available at JLUdata at the University of Giessen (https://jlupub.ub.uni-giessen.de/, accessed on 7 January 2021). The application of GWAInter.R focuses on the detection of possible GxE for SNP markers in a one-step approach. The approach simultaneously enables the estimation of main SNP effects, as well as of SNP by environmental interaction effects, for the two different farming systems—compost and cubicle. Compared with other approaches, it is possible to estimate main SNP effects and interaction effects in one model without splitting the data set. The corresponding statistical model was:

y = Xb + x_snpi_ b_snpi_ + x_interi_ b_interi_ + Zg + e (1)

where y was a vector of observations for the traits LYM, MAC, PMN, sN, bN, MAJOR, MINOR, CNS, COR, AER, and AESC; b was a vector of fixed effects including parity, the general health status of the cow based on somatic cell count levels (with >200,000 cells indicating inferior cow health), farming system (compost or cubicle), farm, herd-test day for trait recording, and the person analyzing the milk samples in the laboratory; x_snpi_ was a vector of genotypes for marker i; b_snpi_ was a regression coefficient for the i*th* SNP (the main SNP effect); x_interi_ was a vector of genotypes for cows; b_interi_ was a regression coefficient of i*th* SNP (the interaction effect); and g was a vector of genetic effects following N(0, Gσ^2^_g_) and contained the polygenic effects, where G was a relationship matrix [30] and σ^2^_g_ was the genetic variance. The G matrix was constructed considering all SNPs, but we excluded the SNP markers from the same chromosome where the candidate SNP was located, i.e., the “leave one chromosome out strategy”; e was the vector of random residual effects following N(0, Iσ^2^_e_), where I was an identity matrix and σ^2^_e_ was the residual variance. X and Z were incidence matrices for b and g, respectively. The “leave one chromosome out approach”, as also implemented in most of the commercial GWAS software packages, was realized by connecting GWAInter.R with the R package “gaston” [31], implying the permanent estimation of variance components per trait and chromosome. That is to say, we gave 29 G matrices (G matrix without chromosome 1, chromosome 2… chromosome 29) to “gaston”, separately for each trait. For the estimation of main SNP and SNP interaction effects, we implemented a generalized least squares approach in GWAInter.R. The *p*-value for each SNP was calculated by applying a test statistic which follows a chi-square distribution (Wald-test). The test statistic for the i*th* main SNP effect was: $\chi_{snpi}^{2}=\frac{{\hat{b_{snpi}}}^{2}}{\mathrm{var}\left(\hat{b_{snpi}}\right)}$, with 1 degree of freedom (df). The test statistic for the i*th* interaction effect was: $\chi_{interi}^{2}=\frac{{\hat{b_{interi}}}^{2}}{\mathrm{var}\left(\hat{b_{interi}}\right)}$, with 1 df.

Significantly associated SNPs were detected according to the Bonferroni corrected significance threshold *p_Bonf_* = 0.05/43,095 SNP markers. Additionally, a less conservative threshold was defined as *p_sugg_* = 0.05/(number of independent SNPs). The number of independent SNPs was 4479 when setting the linkage disequilibrium as ≤0.15 (squared correlation between two SNPs) for consecutive genomic windows including 500 SNPs along the bovine genome.

Potential candidate genes were queried and assigned to associated SNP markers using the current gene annotations from the ENSEMBL and NCBI databases, considering a window 50 kb upstream or downstream from the significantly associated candidate SNP [32,33]. Physiological functions and positions of potential candidate genes were further manually reviewed in the String (version 11) [34], KEGG, and NCBI databases [33,35].

## 3. Results

### 3.1. Genome-Wide Associations

A total of 35 significant (according to the Bonferroni corrected genome-wide significance and less conservative threshold) SNPs for the main effect were detected on 14 different chromosomes for the traits PMN, sN, bN, MAJOR, MINOR, cultural negative, AER, and CNS (Table 1 and Table 2). A subset including eight SNPs surpassed the Bonferroni significance threshold, and 27 SNPs were significant according to the less conservative threshold.

The chromosome including the largest number of significantly (Bonferroni corrected genome-wide significance and less conservative threshold) associated SNPs was chromosome 14. For the cell fractions PMN and sN, the same significant SNPs when considering the less conservative threshold were detected on chromosome 6. One significant (less conservative threshold) SNP on chromosome 28 was significantly associated with bN. For MAJOR, 18 significant (Bonferroni corrected genome-wide significance and less conservative threshold) SNPs were located on chromosomes 1, 2, 4, 5, 11, 15, 17, 18, 22, and 26. Five SNPs exceeded the Bonferroni significance threshold, and 13 exceeded the less conservative threshold. For MINOR, four significantly associated SNPs (according to the less conservative threshold) were detected, and three of them were located on chromosome 14. In addition, for cultural negative, all three significant SNPs (two passed the less conservative threshold and one passed the Bonferroni genome-wide significance) were located on chromosome 14. Four SNPs were identified on chromosomes 4, 5, and 15 for AER. For CNS, three SNPs were detected on chromosome 8.

**Figure 1:**
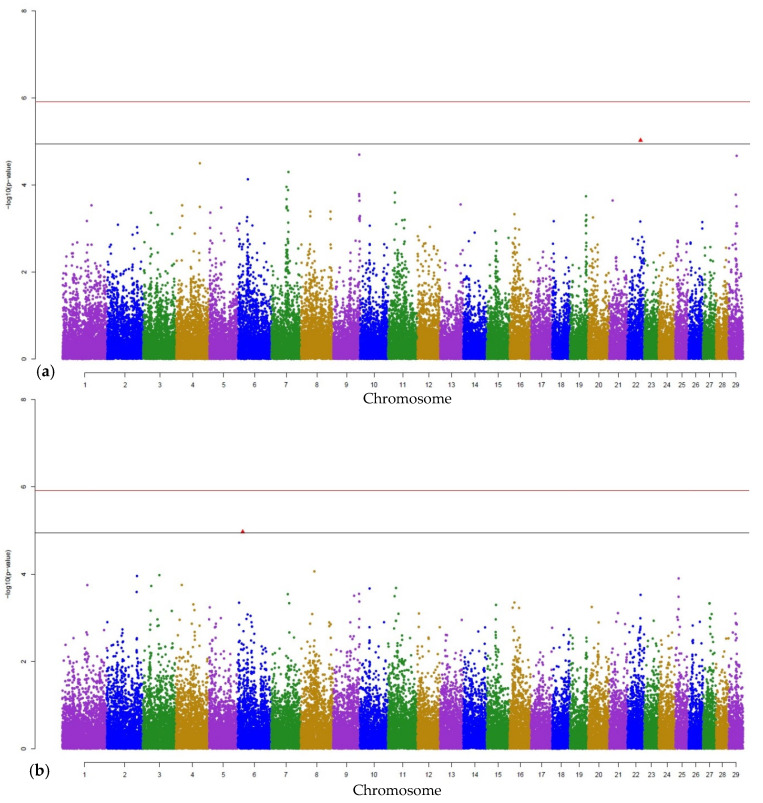
Manhattan plots displaying the GWAS results (*p*-values) for polymorphonuclear neutrophils (PMN): (**a**) indicates the main SNP effect, and (**b**) the interaction effect between SNPs and housing system. The Bonferroni-corrected genome-wide significance (red line) and the less conservative threshold (grey line) (*p_sugg_* = 0.05/4479 = 1.12 × 10^−5^) are shown. Red triangles highlight the significant SNP.

**Figure 2:**
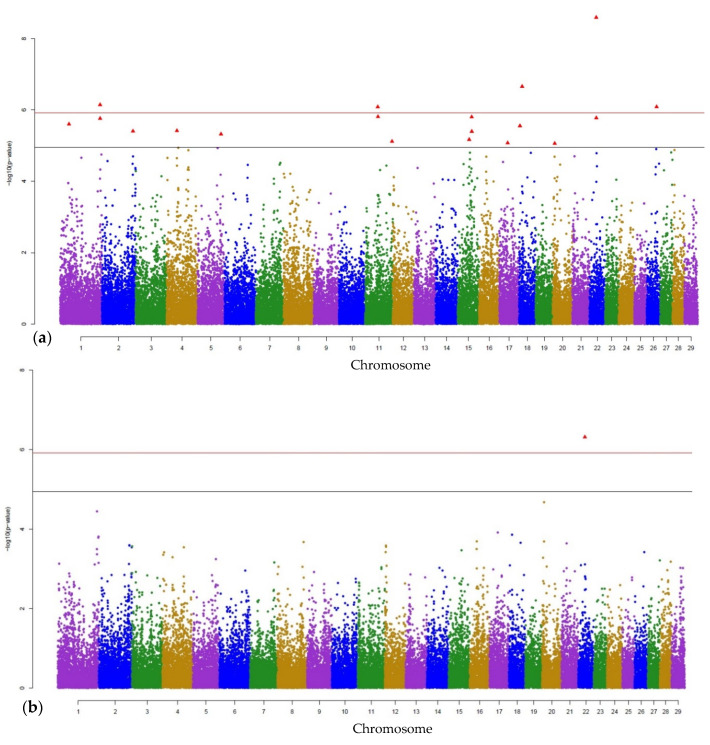
Manhattan plots displaying the GWAS results (p-values) for MAJOR pathogens (*Aerococcus sp.*, *Aesculin hydrolyzing streptococci*, *Candida krusei*, *Enterococcus sp.*, *Escherichia Coli*, *Lactococcus sp.*, *Staphylococcus aureus*, *Streptococcus dysgalactiae*, *Streptococcus uberis*, *mold fungus*, and *Proteus sp.*): (**a**) indicates the main SNP effect, and (**b**) the interaction effect between SNPs and housing system. The Bonferroni-corrected genome-wide significance (red line) and the less conservative threshold (grey line) (*p*_sugg_ = 0.05/4479 = 1.12 × 10^−5^) are shown. Red triangles highlight the significant SNP.

Manhattan plots from the GWAS indicate the most significant SNP (Bonferroni corrected genome-wide significance and less conservative threshold) for PMN as representative of the cell fractions and for MAJOR as representative of the specific mastitis pathogens. Hence, Manhattan plots for main effects and for interaction effects are given in Figure 1a,b, respectively for PMN, and in Figure 2a,b, respectively for MAJOR. Manhattan plots for sN, bN, MINOR, cultural negative, CNS, AER, and AESC are shown in Figure S1.

Regarding SNP interaction effects, significances (Bonferroni corrected genome-wide significance and less conservative threshold) were detected for PMN, MAJOR, MINOR, cultural negative, AER, and AESC. In total, six SNPs were identified as displaying significant interaction effects with the farming system (Table 3), whereas five passed the less conservative threshold. The SNPs were located on chromosomes 3 (three SNPs), 15 (one SNP), and 22 (two SNPs). Only the significant SNP for MAJOR on chromosome 22 surpassed the strict Bonferroni significance threshold.

### 3.2. Gene Annotations

Among the 35 significant SNPs for main effects, 14 were located in different genes (Table 1, Table 2 and Table 3). With regard to the remaining significantly associated SNPs, no genes in close proximity were detected. A significantly associated SNP was identified for PMN and sN on chromosome 6. The annotated *COL25A1* (collagen type XXV alpha 1 chain) gene is a protein-coding gene which is involved in protein digestion and absorption (KEGG entry: bta04974). Functional annotation of the bN candidate gene reveals the involvement of adherent junction (KEGG entry: bta04520), leucocyte transendothelial migration (KEGG entry: bta04670), and bacterial invasion of epithelial cells (KEGG entry: bta05100) for *CTNNA3* (catenin alpha 3). For some potential candidate genes for MAJOR, functional relationships and their roles in biological or functional pathways are not yet known, e.g., for *CRISPLD2* (cysteine rich secretory protein LCCL domain containing 2), *HACL1*(2-hydroxyacyl-CoA lyase 1), and *CAPN5* (caplain 5). Nevertheless, we inferred four other potential candidate genes for MAJOR. *CHL1* (cell adhesion molecule L1 like) on chromosome 22 is a cell adhesion gene playing a role in cell migration and in the suppression of neuronal cell death (KEGG entry: bta04515). *EVA1A* (eva-1 homolog A) on chromosome 11 also mediates autophagy and apoptosis and acts in the regulation of programmed cell death [30]. Further functional annotations of potential candidate genes for MAJOR reveal the involvement of the metabolic pathway (KEGG entry: bta01100) and glycosphingolipid biosynthesis (KEGG entry: bta00601) for *ABO* (alpha 1-3-N-acetylgalactosaminyltransferase and alpha 1-3-galactosyltransferase).

For MINOR, two SNPs were located in the genes *ADAMTSL* (ADAMTS like 1) and *SAMD12* (sterile alpha motif domain containing 12). The same SNP localized in *SAMD12* was detected for the cultural negative group. Likewise, the same SNP in gene *ADAMTSL* was significantly associated with CNS. With regard to CNS, the candidate gene *MLLT3* (MLLT3 super elongation complex subunit) was also identified for CNS, which is involved in the transcriptional misregulation mechanism of cancer (String). Functional annotation of AESC candidate genes indicates the involvement of the cAMP (adenylyl cyclase) signaling pathway for *GRIA4* (glutamate ionotropic receptor AMPA type subunit 4) (KEGG entry: bta04024).

Based on the significant SNP by housing system interaction effects, four different potential candidate genes carrying one significant SNP were detected (Table 3). An associated SNP was identified for PMN on chromosome 22. The annotated *HEMK1* (HemK methyltransferase family member 1) gene is a protein-coding gene which is involved in the methylation of the mitochondrial translation release factor [30]. The SNP located in the *CHL1* gene was significant for the main SNP effect, as well as for the interaction effect between SNPs and farming system. The gene *BTB/POZ 8* (BTB (POZ) domain containing 8) was identified as a possible candidate gene for AER. For AESC, the *TBCEL* (tubulin folding cofactor E like) gene was detected on chromosome 15.

## 4. Discussion

### 4.1. Genome-Wide Association Analyses

The new free walk housing system (compost) implies an enriched bacterial concentration in the bedded pack, depending on management [7]. Thus, the cows are exposed to a higher pathogen burden, which triggers their immune system [7,36]. Due to the higher load on the immune system, the first and memory immune responses change over time, accompanied by alterations to genetic mechanisms [36,37]. Housing-specific mastitis resistances and differing cellular immunological mechanisms suggest specific udder health breeding strategies for alternative farming systems [11,14,37]. Genome-wide associations revealed several potential candidate genes, reflecting the complex nature of udder diseases [12,14]. In our study, among the 41 significant SNPs (Bonferroni corrected genome-wide significance and less conservative threshold), we identified 18 (14 significant SNPs for the main effect and four significant SNPs for the interaction effect) in different genes. However, in the context of udder health, the majority of the detected SNPs and annotated potential candidate genes are reported here for the first time. This is not surprising to us, as our study design, combined with novel udder health indicators, differed widely compared with most previous approaches [11,22]. In addition, compost bedding reflects a challenging and new environmental system, and this study was the first time that GWAS with interaction effects were applied.

Most of the previous genomic udder health studies focused on easier trait definitions such as producer-recorded clinical mastitis or overall somatic cell score. In a study based on specific pathogens [22], significant SNPs on 14 different chromosomes (2, 3, 7, 8, 10, 11, 13, 15, 16, 17, 18, 26, 27, and 28) were reported [22]. Tiezzi et al. [12] used producer records to determine udder health status, and focused only on first parity cows. Significant SNPs were identified on chromosomes 2, 8, 11, 14, 16, 19, 20, 24, and 29. Sørensen et al. [14] estimated genetic correlations among specific mastitis pathogens in the range from 0.45 to 0.77, suggesting that each pathogen should be considered as a specific udder health trait. The lowest correlation of 0.45 was found between *Staphylococcus aureus* and *Escherichia coli*, also explained by the different immune responses of these two pathogens. The highest correlation (0.77) was detected between *Streptococcus dysgalactiae* and *Streptococcus uberis* [14]. Both pathogens belong to the same genus and trigger a similar cascade of immune reactions in the udder [14]. Accordingly, in our GWAS, we identified different significant SNPs (Bonferroni corrected genome-wide significance and less conservative threshold) for the different pathogens, as well as for the differential somatic cell fractions, supporting the quantitative genetic associations [14]. Only four SNPs were significantly associated with two different traits, but these traits were closely related. For example, sN represents a subgroup of PMN, and in such a case overlapping significant SNPs were expected. Similarly, CNS is a fraction of MINOR. Hence, the same SNP located on chromosome 8 was significantly associated with both pathogens. The second significant SNP for MINOR on chromosome 14 was also significant for the cultural negative group. Interestingly, MAJOR displayed a SNP which was significant for both housing systems, as well as for the interaction component.

The different pathogens, which can vary widely between housing systems (compost and cubicle), initiate different immune responses and thus modulate many different genes [38]. These differences are evident especially in the case of *Escherichia coli* and *Staphylococcus aureus* [38]. However, in the last ten years, the diversity of pathogens has shifted from MAJOR to MINOR due to improved stable hygiene management [20,21]. Accordingly, we identified quite high incidences for MINOR, which, in particular, can cause subclinical mastitis. The corresponding immune reactions are less pronounced with weak clinical signs, which complicates the distinction between affected and unaffected animals [39,40]. However, we detected six significant SNPs for the main effect, and one significant SNP by housing system interaction. Consequently, we annotated three potential candidate genes (*ADAMTSL1*, *SAMD12*, and *MLLT3*) for minor pathogens.

### 4.2. Gene Annotations and Functional Pathways

We identified *CTNNA3* as a possible candidate gene. *CTNNA3* is part of the *CTNNA* family with three catenin subtypes (*CTNNA1*, *CTNNA2*, and *CTNNA3*) displaying close functional relationships [41,42]. The catenin subtypes effect a wide range of diseases, such as Alzheimer’s disease or cancer development in humans [42]. CTNNA3 is a key protein of the adherens-junctional complex in epithelial cells and plays a central role in cellular adherence [41]. It also acts as an activator in the MAPK (mitogen-activated protein kinase) pathway [42]. MAPK signaling further regulates the production of target inflammatory genes during clinical mastitis [43].

*EVA1A* is involved in autophagy and programmed cell death [44,45]. Since the invasion of a pathogen into the udder activates phagocytic cells, which afterwards have to be eliminated by apoptosis [46], it is possible that *EVA1A* is also involved in this process.

*HEMK1* was identified as a possible candidate gene. The SNP located in *HEMK1* was significant for the interaction effect, indicating alterations of gene activities with farming system particularities. *HEMK1* acts as a regulator in the JAK-STAT (Janus kinase/signal transducer and activator of transcription) pathway. The JAK-STAT pathway is pivotal for the development and function of the immune system, displaying different mechanisms in different studies [47]. Hence, it is not surprising that *HEMK1* is differently regulated in different housing systems, such as compost and cubicle, due to different environmental stressors. Previous studies demonstrated that immune-related genes such as *IL17*, *IL17F*, and *LIF* indicate GxE [48]. Furthermore, these genes also influence fertility traits, depending on environmental conditions [48].

The *CHL1* gene on chromosome 22, belonging to the L1 family cell adhesion molecule [49], was also detected on the basis of SNP by housing system interactions. In humans, *CHL1* plays a role in diseases triggered by chronic stress [49]. Yang et al. showed that *CHL1* expression in monocytes was significantly downregulated in depressed patients with chronic stress [50]. In addition, these patients showed a reduced number of positive CD19^+^ and CD20^+^ B cells. The downregulation of these two immune cells has a negative effect on the immune system and implies an increased disease susceptibility [50]. Due to improved animal welfare and lower stress levels in compost barns [7], we assume that a possible upregulation of *CHL1* is associated with a positive effect on the immune system and therefore on udder health. A gene expression analysis was conducted in relation to different floor types in Holstein dairy cows [51]. Various types of floor implied different comfort behaviors of cows. Improper lying and walking areas increased stress levels and triggered several cellular processes. In this way, the expression of various genes may be influenced [51,52]. Consequently, a rapid increase in the expression of the gene *HSPA1A* during stress was identified, with ongoing effects on immune system functions [51].

## 5. Conclusions

Based on a novel modelling approach allowing GWAS with SNP by farming system interaction effects, we identified significant main and interaction effects for specific pathogens and cell fractions in milk. The detailed udder health phenotyping strategy, combined with the novel GWAS, contributed to the detection of significant SNPs for PMN, sN, bN, MAJOR, MINOR, cultural negative, CNS, AER, and AESC. For the traits PMN and sN, MINOR and CNS, and MINOR and cultural negative, we identified overlapping significant SNP effects, indicating similar genetic and physiological mechanisms. In this regard, we inferred the potential candidate genes *EVA1A* and *CTNNA3* which are involved in the pathways for autophagy and programmed cell death (*EVA1A*), in the MAPK pathway, and in the adherens-junctional complex in epithelial cells for cellular adherence (*CTNNA3*). Interestingly, for the traits PMN, MAJOR, MINOR, cultural negative, AER, and AESC, we identified significant SNP by farming system interactions, indicating opposite effects of the same SNP markers in cubicle and compost. Annotated potential candidate genes were *CHL1* and *HEMK1*, which are involved in pathways regulating the immune system dependent on stress levels and in the development of the immune system. From a practical perspective, our results suggest farming system-specific breeding strategies in order to improve udder health in both systems. In addition, our new modelling approach considering SNP main and interactions effects simultaneously simplifies the estimations and removes the need to split the data. For future breeding strategies to improve udder health, a very precise definition of mastitis traits is imperative as we identified different genetic mechanisms in response to specific pathogens.

## Figures and Tables

**Table 1:** Genome-wide significant SNPs for the main effect and potential candidate genes associated with the differential somatic cell fractions.

Trait	SNP	Chromosome	Position	*p*-Value SNP	Gene Name
PMN	BTA-110591-no-rs	6	16024226	0.00001080976	*COL25A1*
sN	BTA-110591-no-rs	6	16024226	0.000005703675	*COL25A1*
bN	BTB-00944057	28	22970021	0.000002966474	*CTNNA3*

PMN, polymorphonuclear neutrophils; sN, segmented neutrophils; bN, banded neutrophils; *p*-value: All SNP passed the less conservative threshold (*p_sugg_* = 0.05/4479 = 1.12 × 10^−5^).

**Table 2:** Genome-wide significant SNPs for the main effect and potential candidate genes associated with the specific mastitis pathogens.

Trait	SNP	Chromosome	Position	*p*-Value SNP	Gene Name
MAJOR	ARS-BFGL-NGS-60721	1	35809354	0.000002509339	-
	ARS-BFGL-NGS-26782	1	152561941	0.0000007190281 *	-
	Hapmap23088-BTA-151194	1	152612216	0.000001737926	*HACL1*
	ARS-BFGL-NGS-45691	2	127889562	0.000003947047	-
	ARS-BFGL-NGS-110081	4	41230144	0.000003844456	-
	ARS-BFGL-NGS-29150	5	108921269	0.000004774763	-
	ARS-BFGL-BAC-14274	11	44153677	0.0000008244985 *	*EVA1A*
	Hapmap57340-rs29010501	11	44928962	0.000001551728	-
	ARS-BFGL-NGS−116393	11	104186003	0.00000764746	*ABO*
	ARS-BFGL-NGS−24368	15	44660806	0.000006775498	-
	ARS-BFGL-NGS-67343	15	56153143	0.00000157791	-
	ARS-BFGL-NGS-39731	15	56501007	0.000004006894	*CAPN5*
	ARS-BFGL-NGS-113915	17	32550404	0.000008442227	-
	Hapmap47619-BTA-43853	18	4489809	0.000002801936	-
	Hapmap40333-BTA-10479	18	10989533	0.0000002207883 *	*CHRISPLD2*
	BTA-86068-no-rs	22	26048787	0.000000002563736 *	*CHL1*
	BTA-77184-no-rs	22	26490348	0.000001678371	-
	ARS-BFGL-NGS-39928	26	38508625	0.0000008220506 *	-
MINOR	ARS-BFGL-NGS-27512	8	25393606	0.0000079641	*ADAMTSL1*
	UA-IFASA-8766	14	42871327	0.000001380271	-
	Hapmap47921-BTA-34862	14	45867755	0.00001113587	*SAMD12*
	ARS-BFGL-NGS-112964	14	68578807	0.000002708568	-
cultural negative	BTB-01709715	14	44933472	0.000002761847	-
	Hapmap47921-BTA-34862	14	45867755	0.000003396697	*SAMD12*
	ARS-BFGL-BAC-23102	14	68228943	0.0000009770646 *	-
AER	ARS-BFGL-NGS-93391	4	8794043	0.000003244757	-
	BTB-00909994	5	3684232	0.00001095756	-
	ARS-BFGL-NGS-40368	15	2048607	0.000009708123	*GRIA4*
	ARS-BFGL-NGS-9407	15	2578791	0.000000220117 *	-
CNS	Hapmap51393-BTA-113111	8	23814719	0.000004550739	*MLLT3*
	BTA-103194-no-rs	8	24224606	0.000009541055	-
	ARS-BFGL-NGS-27512	8	25393606	0.0000005630255 *	*ADAMTSL1*

MAJOR, *Aerococcus sp.*, *Aesculin hydrolyzing streptococci*, *Candida krusei*, *Enterococcus sp.*, *Escherichia Coli*, *Lactococcus sp.*, *Staphylococcus aureus*, *Streptococcus dysgalactiae*, *Streptococcus uberis*, *mold fungus*, and *Proteus sp.*; MINOR, *Coagulase-negative staphylococci* and *Corynebacterium sp.*; AER, *Aerobic bacilli*; CNS, *Coagulase-negative staphylococci*; * Bonferroni-corrected genome-wide significance, otherwise less conservative threshold (*p*_sugg_ = 0.05/4479 = 1.12 × 10^−5^). “-” no gene was found inside the 50kb window.

**Table 3:** Genome-wide significances for interactions between SNPs and housing systems and annotated potential candidate genes for differential somatic cell fractions and for mastitis pathogens.

Trait	SNP	Chromosome	Position	*p*-Value Interaction	Gene Name
GGPMN	ARS-BFGL-NGS-16330	22	49768282	0.000009423205	*HEMK1*
MAJOR	BTA-86068-no-rs	22	26048787	0.0000004836461 *	*CLH1*
MINOR	ARS-BFGL-NGS-111815	3	118644571	0.000004458816	-
cultural negative	ARS-BFGL-NGS-111815	3	118644571	0.000002214425	-
AER	INRA-611	3	51240435	0.00000619058	*BTB 8*
AESC	BTB-00591978	15	31666125	0.000009842745	*TBCEL*

PMN, *polymorphonuclear neutrophils*; MAJOR, *Aerococcus sp.*, *Aesculin hydrolyzing streptococci*, *Candida krusei*, *Enterococcus sp.*, *Escherichia Coli*, *Lactococcus sp.*, *Staphylococcus aureus*, *Streptococcus dysgalactiae*, *Streptococcus uberis*, *mold fungus*, and *Proteus sp*.; MINOR, *Coagulase-negative staphylococci* and *Corynebacterium sp.*; AER, *Aerobic bacilli*; AESC, *Aesculin hydrolysing streptococci*; * Bonferroni-corrected genome-wide significance, otherwise less conservative threshold (*p*_sugg_ = 0.05/4479 = 1.12 × 10^−5^).“-” no gene was found inside the 50 kb window.

## Supplementary Materials

The following are available online at https://www.mdpi.com/article/10.3390/ani11061839/s1, Figure S1: Manhattan plots displaying the GWAS results (*p*-values) for the main SNP effects for: (a) segmented neutrophils, (c) banded neutrophils, (e) MINOR pathogens (*Coagulase-negative staphylococci* and *Corynebacterium sp*.), (g) cultural negative, (i) *Aerobic bacilli*, (k) *Aesculin hydrolyzing streptococci*, and (m) *Coagulase-negative staphylococci.* Manhattan plot displaying the GWAS results (*p*-values) for the interaction of the SNP and housing system effects for: (b) segmented neutrophils, (d) banded neutrophils, (f) MINOR pathogens (*Coagulase-negative staphylococci* and *Corynebacterium sp*.), (h) cultural negative, (j) *Aerobic bacilli,* (l) *Aesculin hydrolyzing streptococci*, and (n) *Coagulase-negative staphylococci.* Bonferroni-corrected genome-wide significance (red line) and less conservative threshold (grey line) (*p_sugg_* = 0.05/4479 = 1.12 × 10^−5^) are shown. Red triangles highlight the significant SNP.
