# Supplementary material for: Genome-Wide Associations for Microscopic Differential Somatic Cell Count and Specific Mastitis Pathogens in Holstein Cows in Compost-Bedded Pack and Cubicle Farming Systems
Source: Animals (Basel). 2021 Jun 21;11(6):1839. doi: 10.3390/ani11061839 (PMC8234204; doi:10.3390/ani11061839)
Supplement: Supplementary file 1 [file animals-11-01839-s001.zip › animals-1228729-supplementary.pdf]

animals

MDPI

Supplementary Figure 1:

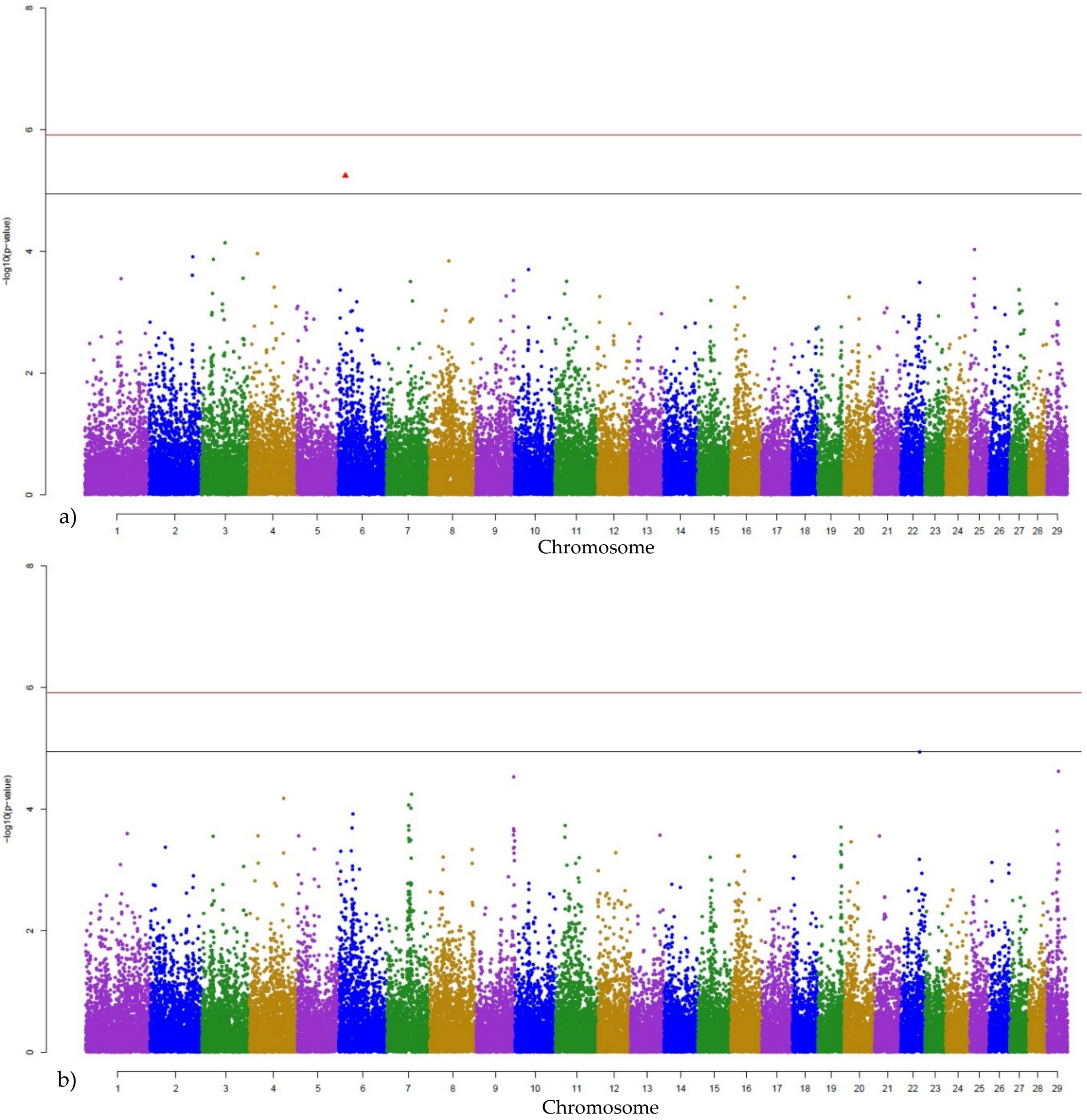

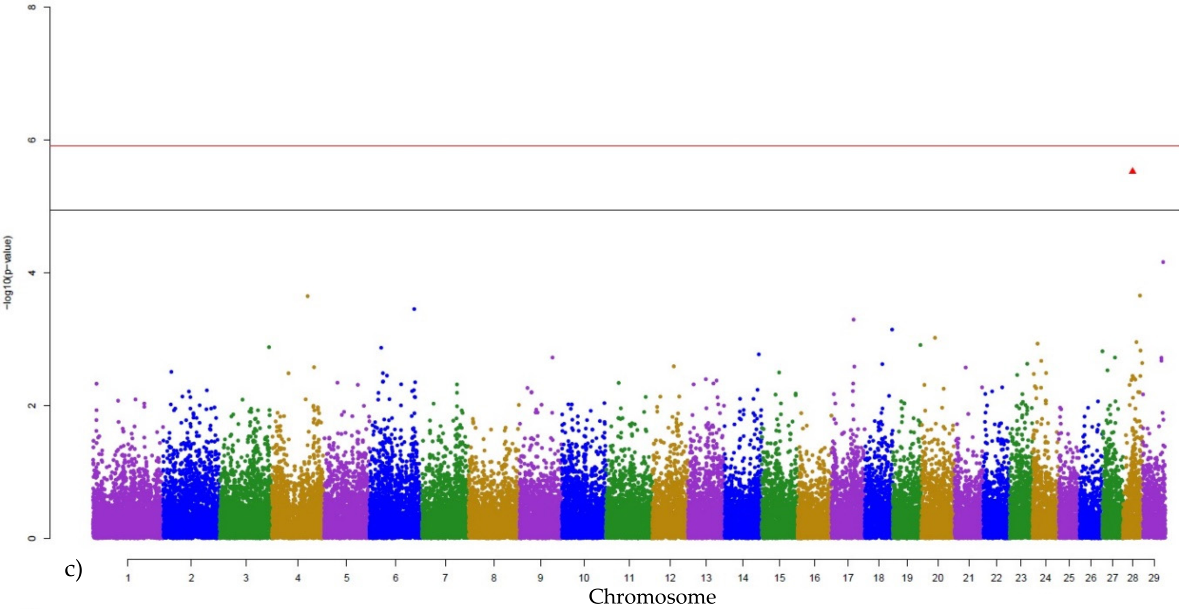
-log10(p-value)
Chromosome
c)

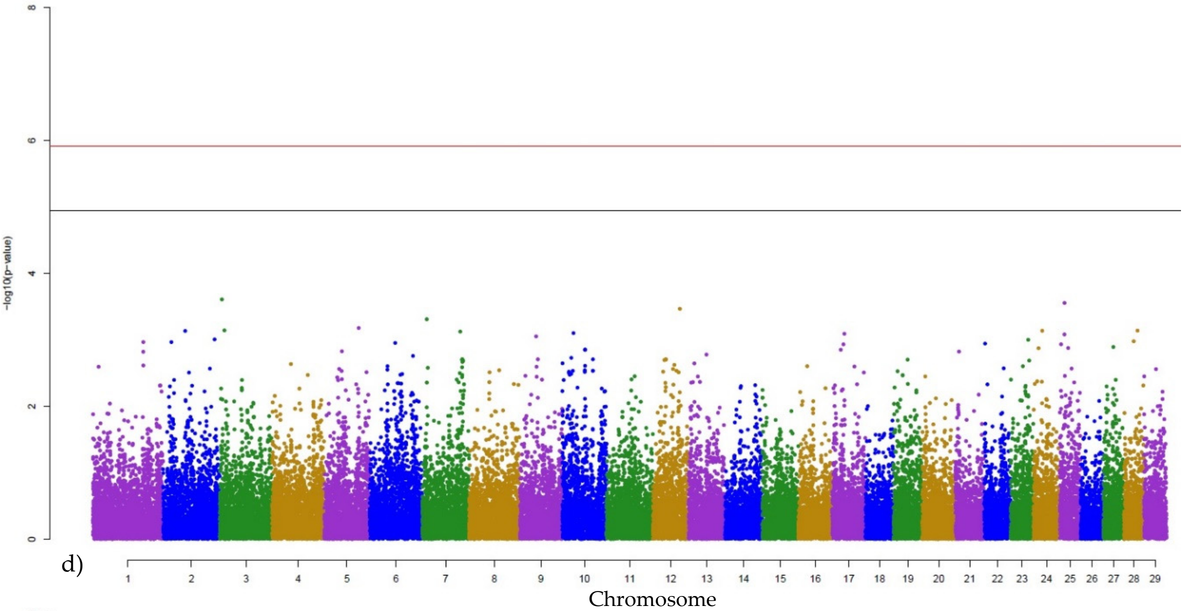
-log10(p-value)
Chromosome
d)

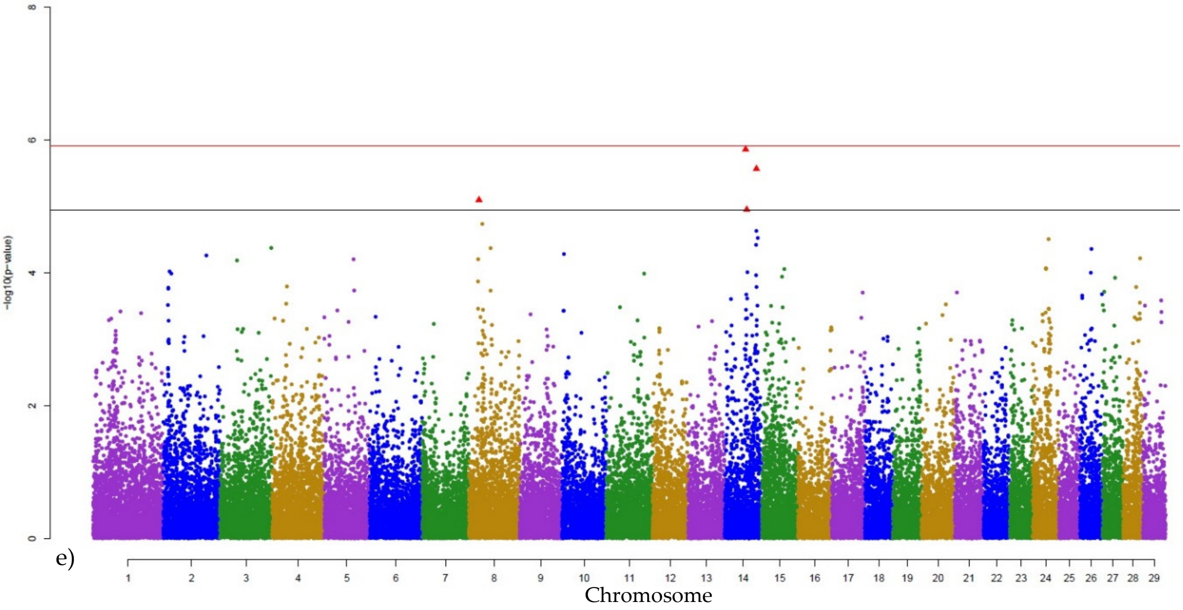
-log10(p-value)
Chromosome
e)

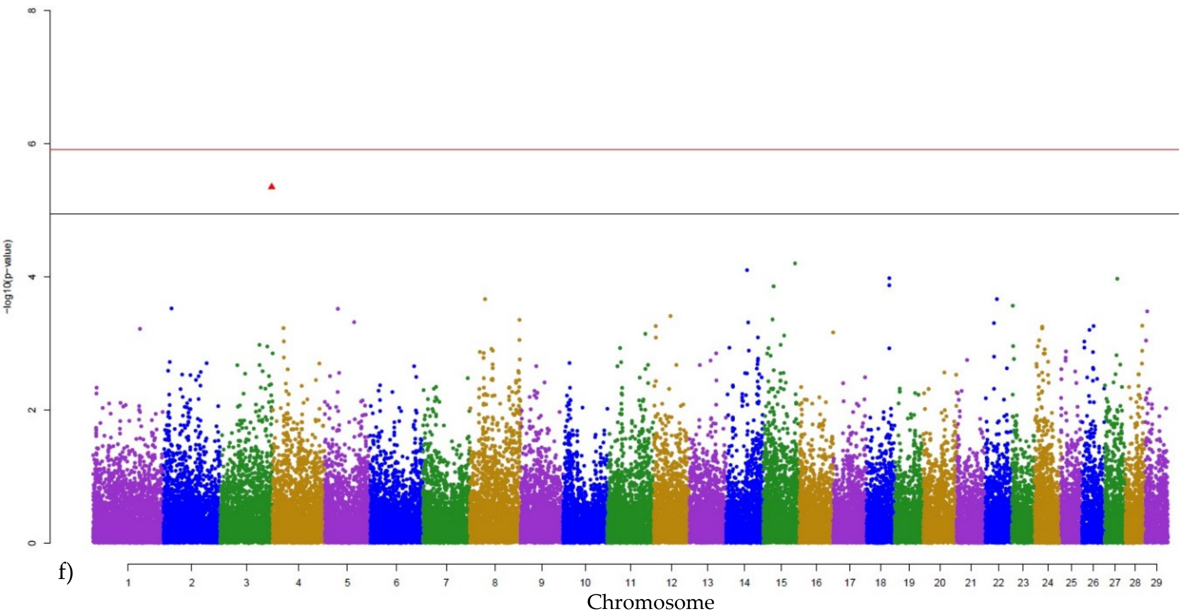
-log10(p-value)
Chromosome

f)

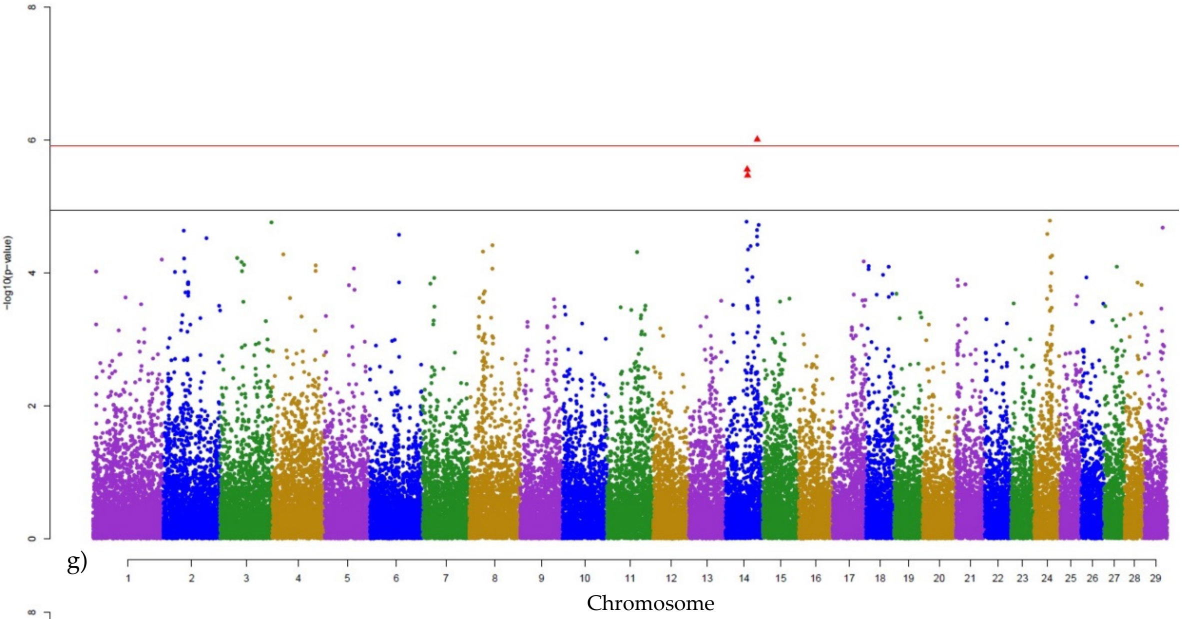
-log10(p-value)
Chromosome

g)

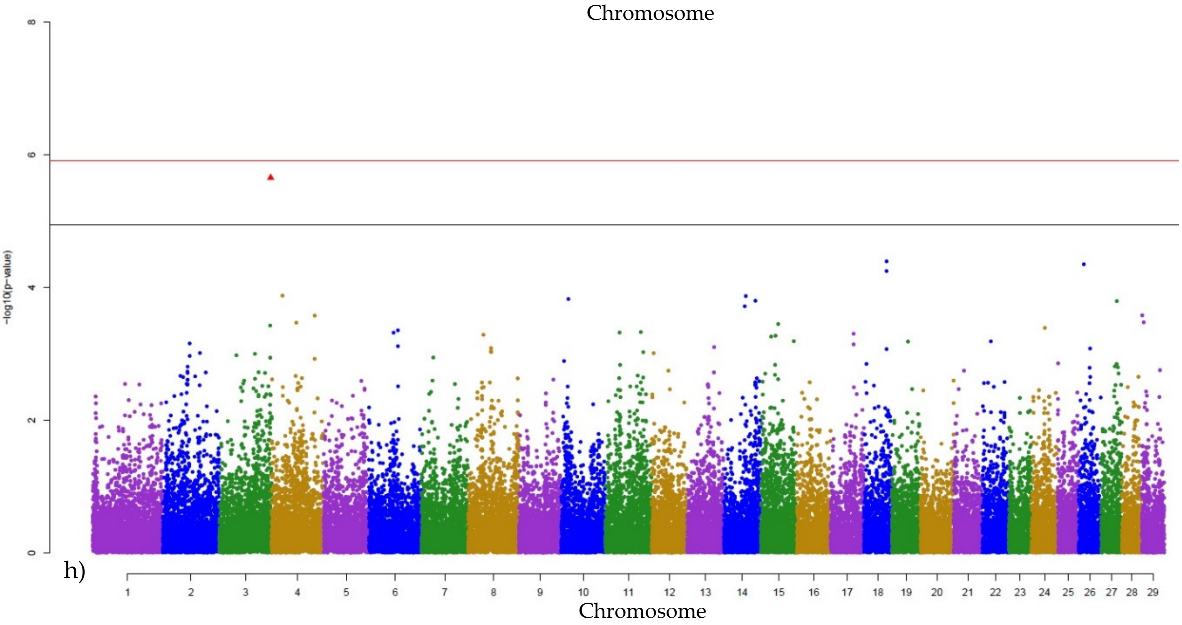
-log10(p-value)
Chromosome

h)

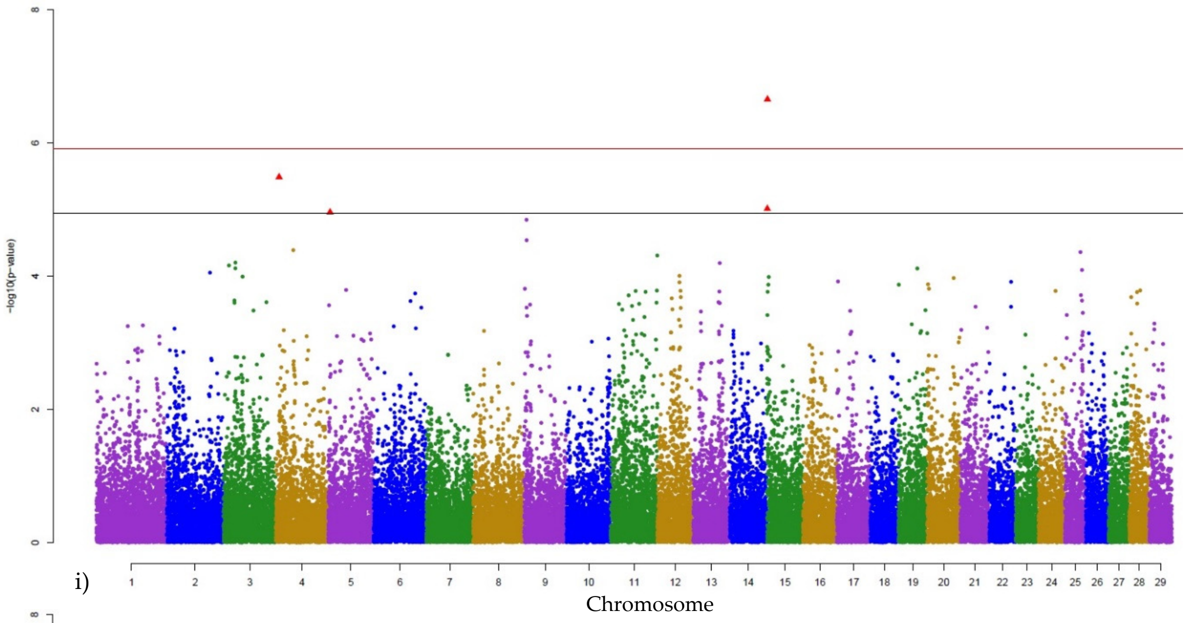

-log10(p-value)
0
2
4
6
8
1 2 3 4 5 6 7 8 9 10 11 12 13 14 15 16 17 18 19 20 21 22 23 24 25 26 27 28 29
Chromosome

i)

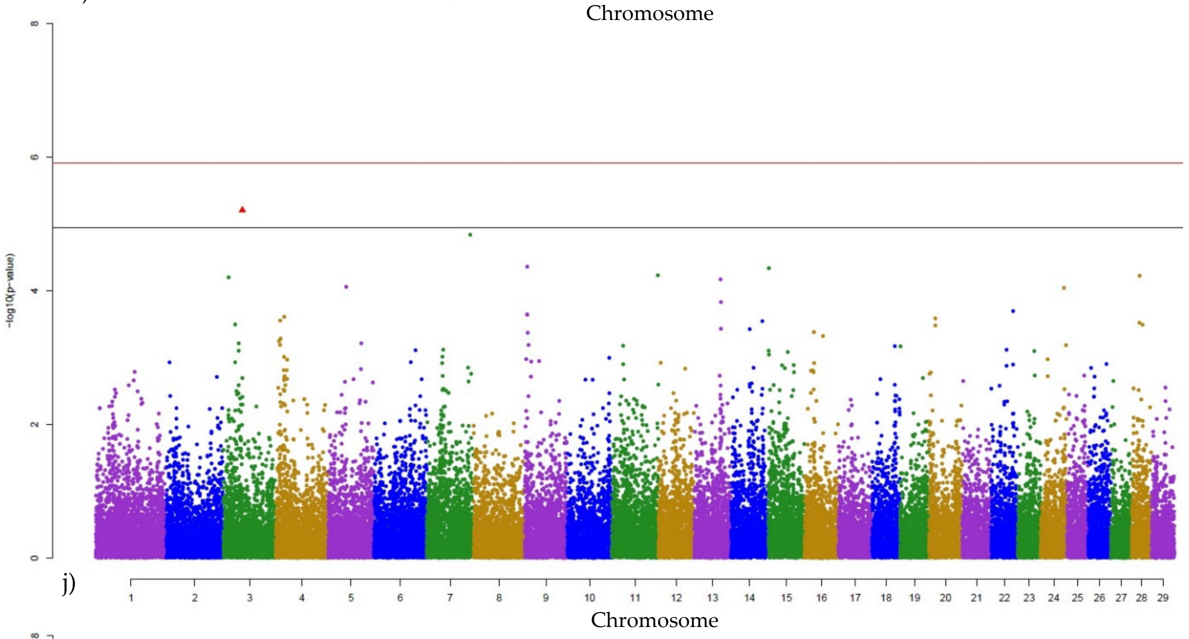

-log10(p-value)
0
2
4
6
8
1 2 3 4 5 6 7 8 9 10 11 12 13 14 15 16 17 18 19 20 21 22 23 24 25 26 27 28 29
Chromosome

j)

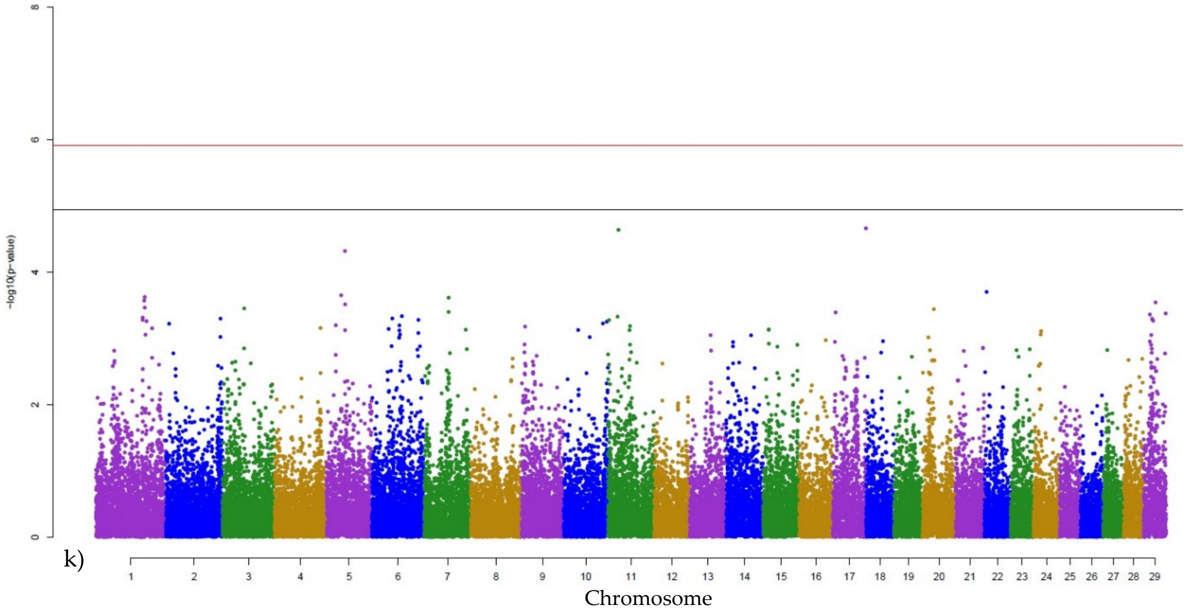

-log10(p-value)
0
2
4
6
8
1 2 3 4 5 6 7 8 9 10 11 12 13 14 15 16 17 18 19 20 21 22 23 24 25 26 27 28 29
Chromosome

k)

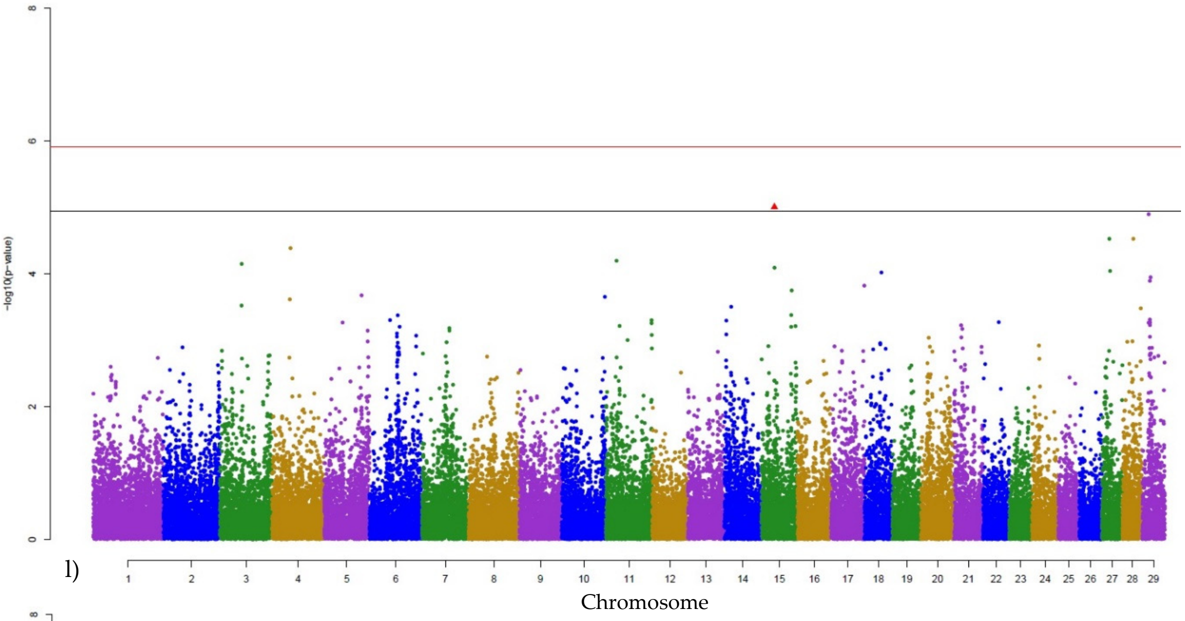
-log10(p-value)
0 2 4 6 8
1 2 3 4 5 6 7 8 9 10 11 12 13 14 15 16 17 18 19 20 21 22 23 24 25 26 27 28 29
Chromosome

l)

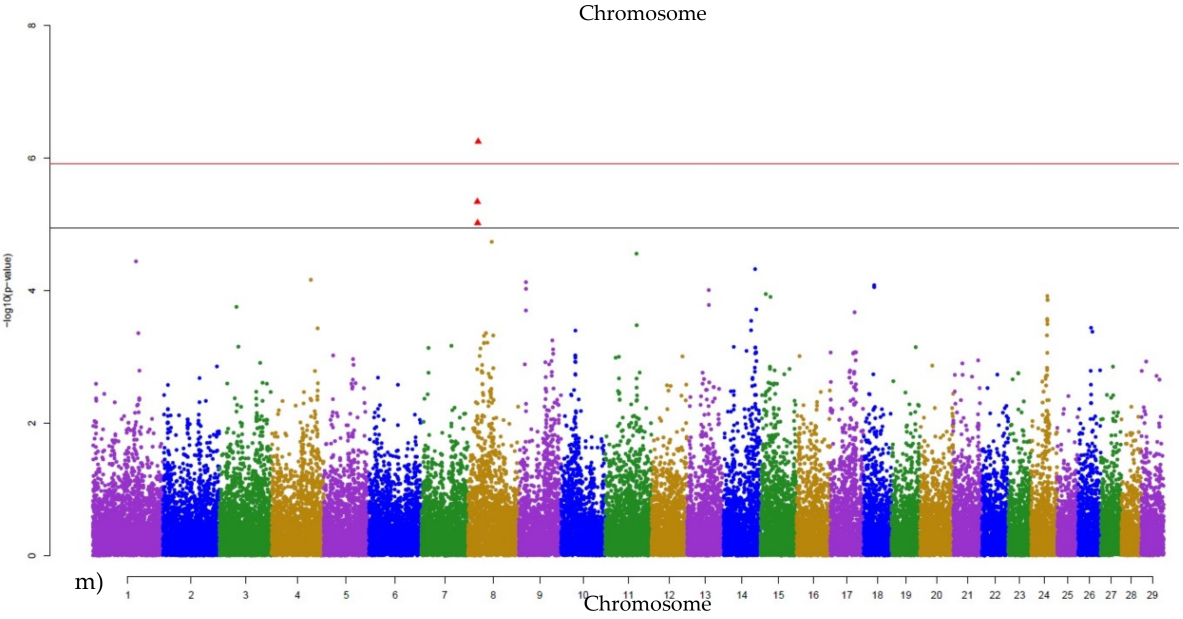
-log10(p-value)
0 2 4 6 8
1 2 3 4 5 6 7 8 9 10 11 12 13 14 15 16 17 18 19 20 21 22 23 24 25 26 27 28 29
Chromosome

m)

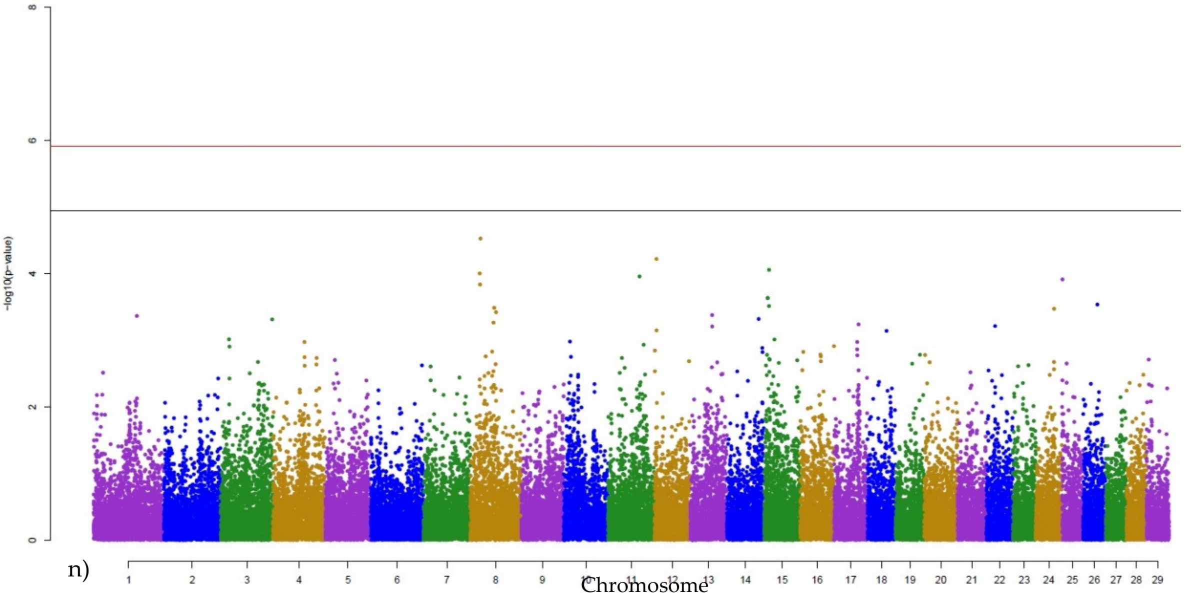
-log10(p-value)
0 2 4 6 8
1 2 3 4 5 6 7 8 9 10 11 12 13 14 15 16 17 18 19 20 21 22 23 24 25 26 27 28 29
Chromosome

n)

Supplementary Figure 1. Manhattan plot displaying the GWAS results (p-values) of the main SNP effects for a) segmented neutrophils, c) banded neutrophils, e) MINOR pathogens (*Coagulase-negative staphylococci* and *Corynebacterium sp*.), g) cultural negative, i) *Aerobic bacilli, k) Aesculin hydrolyzing streptococci* and m) *Coagulase-negative staphylococci.* Manhattan plot displaying the GWAS results (p-values) of interaction of the SNP effects and the effects of cows in the housing systems for b) segmented neutrophils, d) banded neutrophils, f) MINOR pathogens (*Coagulase-negative staphylococci* and *Corynebacterium sp*.), h) cultural negative, j) *Aerobic bacilli, l) Aesculin hydrolyzing streptococci* and n) *Coagulase-negative staphylococci*. Bonferroni-corrected genome-wide significance (red line) and less conservative threshold (grey line) (P_{sugg} = 0.05/4479 = 1.12 × 10^{-5}) are shown. Red triangles highlight the significant SNP.
